# Circular RNA CircSLC8A1 contributes to osteogenic differentiation in hBMSCs via CircSLC8A1/miR‐144‐3p/RUNX1 in periprosthetic osteolysis

**DOI:** 10.1111/jcmm.17633

**Published:** 2022-12-20

**Authors:** Boning Yang, Yu Qin, Ao Zhang, Penghao Wang, Hua Jiang, Yunyi Shi, Guanchao You, Dianlin Shen, Shenghui Ni, Lei Guo, Ying Liu

**Affiliations:** ^1^ Department of Orthopedic Surgery, First Affiliated Hospital China Medical University Shenyang Liaoning China; ^2^ Department of Nursing, First Affiliated Hospital China Medical University Shenyang Liaoning China

**Keywords:** CircSLC8A1, hBMSCs, miR‐144‐3p, osteogenic differentiation, osteopontin, periprosthetic osteolysis, RUNX1

## Abstract

Circular RNAs (circRNAs) are often found in eukaryocyte and have a role in the pathogenesis of a variety of human disorders. Our related research has shown the differential expression of circRNAs in periprosthetic osteolysis (PPOL). However, the involvement of circRNAs in the exact process is yet unknown. CircSLC8A1 expression was evaluated in clinical samples and human bone marrow mesenchymal stem cells (hBMSCs) in this investigation using quantitative real‐time PCR. In vitro and in vivo studies were conducted to explicate its functional role and pathway. We demonstrated CircSLC8A1 is involved in PPOL using gain‐ and loss‐of‐function methods. The association of CircSLC8A1 and miR‐144‐3p, along with miR‐144‐3p and RUNX1, was predicted using bioinformatics. RNA pull‐down and luciferase assays confirmed it. The impact of CircSLC8A1 in the PPOL‐mouse model was also investigated using adeno‐associated virus. CircSLC8A1 was found to be downregulated in PPOL patients' periprosthetic tissues. Overexpression of CircSLC8A1 promoted osteogenic differentiation (OD) and inhibited apoptosis of hBMSCs in vitro. The osteogenic markers of RUNX1, osteopontin (OPN) and osteocalcin (OCN) were significantly upregulated in hBMSCs after miR‐144‐3p inhibitor was transferred. Mechanistic analysis demonstrated that CircSLC8A1 directly bound to miR‐144‐3p and participated in PPOL through the miR‐144‐3p/RUNX1 pathway in hBMSCs. Micro‐CT and quantitative analysis showed that CircSLC8A1 markedly inhibited PPOL, and osteogenic markers (RUNX1, OPN and OCN) were significantly increased (P<0.05) in the mice model. Our findings prove that CircSLC8A1 exerted a regulatory role in promoting osteogenic differentiation in hBMSCs, and CircSLC8A1/miR‐144‐3p/RUNX1 pathway may provide a potential target for prevention of PPOL.

## 1 INTRODUCTION

Periprosthetic osteolysis (PPOL) is still a major limiting factor in the longevity of prosthetic joints. Long time follow‐up dates (about 15–20 years) point out that approximately 55% of patients after total hip arthroplasty (THA) have accepted revision surgery leading by PPOL; and nearly 31% of total knee arthroplasty (TKA) patients suffer the same situation.^1^ Due to its serious complications and complex procedure, revision surgery is not only a huge challenge for patients but also a tough decision for surgeons. Recent studies indicate the main pathological basis is attribute to wear particles generated by the prosthesis–bone interface. These particles induce a chronic inflammation and destroy the bone homeostasis.^2^ Finally, it leads to periprosthetic osteolysis.

Circular RNAs (circRNAs), which have a covalently closed single‐stranded stable structure, assume important roles in cellular function.^3^ With the convergence of sophisticated sequencing and bioinformatics, a considerable number of circRNAs have been observed in multiple musculoskeletal pathologies.^4^ For example, Liu et al.^5^ reported that Circ‐CER could be a therapeutic target for osteoarthritis (OA) via manipulating MMP13 expression through the Circ‐CER/miR‐136/MMP13 axis; latest research by Wen et al.^6^ suggests that has_circ_0001200, has_circ_0001566, has_circ_0003972, and has_circ_0008360 might influence the onset and progression of rheumatoid arthritis (RA) and probably serve as predictive biomarkers for RA diagnosis. Intervertebral disc degeneration (IDD) seems to be inhibited by has_circ_0001658 in several investigations. Treatment for IDD may also be enhanced by improving the expression of has_circ_0001658, which could compete with miR‐181c‐5p and downregulated FAS to inhibit the apoptosis of human neural progenitor cells (hNPCs).^7^ At the molecular level, circRNAs have been determined to have important functions as follows: (i) compete with endogenous RNAs or miRNA sponges,^8^ (ii) act as transcriptional regulators,^9^ (iii) bind to proteins,^10^ and (iv) participate in protein translation.^11^

However, few researches concern about the expression pattern, biological function and pathway of circRNAs in PPOL. Our previous study analysed the expression patterns of circRNA in PPOL and normal tissues, which were obtained through high‐throughput sequencing technologies. It was observed that one upregulated circRNA and five downregulated circRNAs were statistically significant, indicating that these six circRNAs should be investigated further.^12^ Based on the outcome of RNA‐seq, we finally picked CircSLC8A1, one of the dramatically downregulated circRNAs originating from the SLC8A1 gene with the circBase ID of has_circ_0000994. Following that, we discovered direct evidence that CircSLC8A1 modulates bone formation by targeting miR‐144‐3p/RUNX1. Our findings have given us new insight into the prognosis of PPOL and its available therapeutic target.

## 2 MATERIALS

### 2.1 Clinical samples

Clinical samples were donated by patients after written informed consent who underwent orthopaedic surgery. Patients must have clinical manifestations and show a distinct line around the prosthesis and a visible change in imaging findings to be selected for inclusion in this study. Six individuals with PPOL who underwent revision surgery had their periprosthetic tissues obtained and studied. All of the samples had been frozen in liquid nitrogen and preserved at −80°C until RNA extraction. Another six paired samples, which were harvested from patients who suffered femoral neck fractures during primary total hip arthroplasty (THA), were decided to be used as controls. All procedures in our studies were granted permission by the First Affiliated Hospital's Research Ethics Committee of China Medical University. Clinical samples were tested for the expression of CircSLC8A1, miR144‐3p and RUNX1 using qRT‐PCR and Western blotting.

### 2.2 RNA extraction and quantitative real‐time polymerase reaction (qRT‐PCR)

TRIzol reagent (Thermofisher) was used to extract total RNAs, and TriPure Isolation Reagent (Roche) was used to isolate genomic DNA (gDNA), all as recommended by the manufacturer's instructions. RNA samples were tested using the Nanodrop 2000 (Thermo Fisher Scientific), which was acquired to determine their concentration and purity. Two different reverse transcriptions of circRNA and mRNA were performed using the 2× Power Taq PCR MasterMix (BioTeke). The TaqMan MicroRNA Reverse Transcription Kit (Thermo Fisher) was used to accomplish the reverse transcriptions of miRNAs in this study. SYBR Green (Solarbio) and QuantStudio3 (Thermo Fisher) were used for quantitative real‐time PCR, with each sample being tested three times. In order to examine the relative quantity of circRNA, miRNA, and mRNA expression, the 2^−ΔΔCT^ approach was adopted and normalized by GAPDH or U1. The primers were listed in Table 1.

**TABLE 1:** Real‐time PCR primers

Gene name	Forward primer	Reverse primer
CircSLC8A1	5'‐ TTGAGGACACTTGTGGAGA −3'	5'‐GACTCACAGTAACTAACAGATG‐3'
mmu_circ_0000823	5'‐TGAGGACACCTGTGGAGA −3'	5'‐ CCAGAGCTACCAGACGAA −3'
miR‐144‐3p	5'‐AGTAGATATGACATGCGCGCG‐3'	5’‐AGTGCAGGGTCCGAGGTATT‐3’
RUNX1	5'‐ CATCGCTTTCAAGGTGGTGG‐3'	5'‐ ATGGCTGCGGTAGCATTTCT −3'
OPN	5'‐CTCCATTGACTCGAACGACTC‐3'	5'‐CAGGTCTGCGAAACTTCTTAGAT‐3'
OCN	5'‐GGCGCTACCTGTATCAATGG‐3'	5'‐GTGGTCAGCCAACTCGTCA‐3'
GAPDH	5'‐AGATCCCTCCAAAATCAAGTGG‐3'	5'‐GGCAGAGATGATGACCCTTTT‐3'

### 2.3 Nucleic acid electrophoresis

After extracting gDNA and total RNA, divergent primers and convergent primers were used to reverse‐transcripted CircSLC8A1 were obtained from Genewiz company. GAPDH was used as a linear RNA control. The products were subjected to SDS‐PAGE gel electrophoresis with TBE running buffer. The UV shadow method was used to observe nucleic acid.

### 2.4 Cell culture and identification

Human bone marrow mesenchymal stem cells (hBMSCs) were supplied by the 307‐Ivy Translational Medicine Center. Complete culture medium (CM‐H166, Procell China) had been used to grow the cells at 37°C in 95% humidity with 5% CO_2_ and was replenished every 2 days. Cells were passaged and digested with 0.25 trypsin–EDTA (Thermo Fisher) when they reached 80%–90% confluence. The subsequent studies were performed employing hBMSCs from the third passage. For osteogenic induction, hBMSCs were grown in an osteogenic differentiation medium (HUXMA‐90021; Cyagen Biosciences) that included 20 ml of osteogenic differentiation foetal bovine serum, and refreshed every 3 days.

### 2.5 Cell transfection

For silencing CircSLC8A1, small interfering RNA targeting CircSLC8A1 (siRNA), and siRNA‐negative control siRNA (si‐NC), miR‐144‐3p inhibitors, miR‐144‐3p mimics and their negative control were synthesized by GenePharma. pcDNA 3.1 vectors (Beierbo) were purchased to construct CircSLC8A1 and RUNX1 overexpression vectors (oe‐CircSLC8A1 and oe‐RUNX1). Briefly, hBMSCs were transplanted onto the six‐well plates and cultivated until they reached an 80% confluence rate, at which stage they were collected and used in further experiments. Transfecting cells using Lipofectamine 2000 reagent (Thermo Fisher) was performed in accordance with the manufacturer's instructions. The siRNA sequences were listed in Table 2.

**TABLE 2:** si‐RNA sequence of CircSLC8A1

Gene name	RNA
Si‐RNA1	AATTGTTAGGTTGTGACAGTT
Si‐RNA2	GATGAAATTGTTAGGTTGTGA
Si‐RNA3	AAATTGTTAGGTTGTGACAGT

### 2.6 Flow cytometry analysis

Apoptosis was detected using the Annexin V‐APC/ 7‐AAD detection Kit (Procell China). The treated cells were digested and centrifuged at 300 *g* for 5 min before being rinsed in cold PBS and resuspended in a solution containing 500 L diluted 1Annexin V Binding Buffe, 5 L Annexin V‐APC, and 5 L 7‐AAD staining solution. Cells were incubated at 37°C in dark for 15–20 min. This was followed by an analysis of the labelled cells using the Calibur Flow Cytometer's Fluorescence‐activated cell sorting (FACS) technology (Becton Dickinson).

### 2.7 Immunofluorescence (IF)

hBMSCs were incubated in a glass bottom culture dish (35 mm in diameter). A total of 4% paraformaldehyde were used to fix the cells and penetrated with 0.3% Triton X‐100 for 15 min, then 1% bovine serum albumin was used to block the cells for 30 min, followed by the incubation of rabbit anti‐human RUNX1 (ab240639; Abcam) at 4°C overnight. Finally, the treated cells had been washed three times with 4°C PBS and incubated with goat antirabbit secondary antibodies conjugated fluorescent cy5 at 37°C for 30 min (ab150077; Abcam) for IF.

### 2.8 Bioinformatics analysis

The circular RNA interactome database (https://circinteractome.nia.nih.gov/) predicted Circ's miRNA targets. The mouse homologous circRNA was obtained through the circRNA online database circBase (https://www.circbase.org/), and the DNAMAN system was used to match CircSLC8A sequences between mouse and human (LynnonBiosoft). TargetScan (http://www.targetscan.org/vert71/) was used to identify probable target genes for miR‐144‐3p.

### 2.9 Pull‐down assay

Streptavidin‐biotin and magnetic beads were pretreated with 1% RNase free BSA and 0.5% g/L yeast tRNA at 4°C for 3 h, then biotin‐labelled probe of CircSLC8A1 and a control sequence were mixed and incubated. A total of 10^7^ hBMSCs were lysed with 750 μl lysis buffer including 20 mmol/L Tris HCl (pH 7.5), 200 mmol/L NaCl, 5 mmol/L MgCl_2_, 6 × 10^4^ U/L RNase inhibitor, 1 mmol/L DTT and protein inhibitor, and then 1000 *g* centrifuged at 4°C for 5 min. qRT‐PCR was used to detect miR‐144‐3p expression after circRNA compounds were extracted.

### 2.10 Luciferase activity assays

The 3′‐untranslated region (3′‐UTR) of CircSLC8A1 and RUNX1 were integrated into pmirGLO plasmids (Genepharma) to form luciferase reporter vectors: pmirGLO‐ CircSLC8A1‐wt and pmirGLO‐RUNX1‐wt. The mutant vectors were manufactured by constructing mutant target sequences and inserting them into pmirGLO to get pmirGLO‐CircSLC8A1‐mut and pmirGLO‐RUNX1‐mut. HEK‐293 T cells had been cultured in 12‐well plates and reached 80% confluence, then cotransfected these plasmids with miR‐144‐3p mimics or miR‐144‐3p NC using Lipofectamine® 2000 (Invitrogen) at 37°C with 5% CO_2_ for 5 h. The supernatant of transfection medium was removed and replaced with DMEM medium (Gibco) with 10% serum. A dual‐luciferase reporter assay kit (Promega) was utilized to detect the luciferase activity after 24 h. Relative luciferase activity was estimated by the ratio between firefly and renilla luciferase.

### 2.11 RNA fluorescent in situ hybridization

Following cotransfection with CircSLC8A1 vectors and miR‐144‐3p mimics, the fluorescence in situ hybridization (FISH) assay was carried out in hBMSCs. The hybridization was performed employing Cy3‐labelled CircSLC8A1 and FAM‐labelled and miR‐144‐3p mimics, following the manufacturer's instructions. DAPI was used to counterstain the cell nucleus. An IX‐53 microscope was used to capture the pictures (Olympus).

### 2.12 Western blot

Total protein was extracted by using RIPA Lysis Buffer (Beyotime) in a cryogenic refrigerated centrifuge (H‐2050R, Cence) at 4°C for 5 min. Protein determination was measured by the BCA Protein Assay Kit (Beyotime). Loading solutions were prepared for standby by diluting the protein sample with loading buffer and PBS, then heating it for 5 min in water bath. Proteins were separated by 5% SDS‐PAGE and transferred to polyvinylidene fluoride (PVDF) membranes. Polyvinylidene fluoride membranes were incubated overnight at 4°C with antihuman RUNX1 (1:1000; Abcam), OPN (1:1000; Proteintech), and OCN (1:500; Proteintech) antibodies at the appropriate dilution concentrations, followed by 2 h at 37°C with the secondary antibody. The antibody to GAPDH (1:2500; Bioss) was used as a control. The blots were detected by ECL chemiluminescent reagent (Millipore) and a Gel‐Pro‐Analyzer system.

### 2.13 Alizarin red S staining (ARS)

After osteogenic incubation for 14 days, hBMSCs were fixed in absolute ethanol for 30 min and washed twice with PBS, and then stained with 0.2%, pH 4.1–4.3, Alizarin red S (Yeasen), and incubated at 37°C with slight shaking, according to the manufacturer's procedure. The cells should be washed three times with PBS to remove dye that was not fully attached. After excess double distilled water was removed, absorbance was measured at 420 nm using a Varioskan LUX (Thermo).

### 2.14 Wear particle preparation

Titanium alloy particles (DK) with an average diameter of approximately 40 nm were used in this project. Particles were washed twice with 75% ethanol and soaked in anhydrous ethanol for 24 h to eliminate endotoxin. The Limulus Amebocyte Lysate Assay was used to evaluate endotoxin levels after being dried in a desiccator.

### 2.15 Animal experiments

Further in vivo tests were performed on 12‐week‐old adult male C57BL/6 mice. Pin‐implantation surgery was used to create a mouse PPOL model as follows: for anaesthesia, 30 mg/kg of 10% chloral hydrate was administered intraperitoneally. A medial parapatellar incision of the right knee joint with a length of 5 mm was chosen. The tibial plateau was exposed after the patella was moved aside, and a proximal 6 mm of the tibial intramedullary canal was drilled through the middle of the tibial plateau with a 0.8 mm dental drill.^13^ Before insertion of the pin implant during surgery, a dose of 4 × 10^4^ titanium particles suspension was injected into the tibia canal to imitate prosthetic wear. Adeno‐associated virus (AAV) was chosen to overexpress CircSLC8A1 in vivo through articular injection. AAV vectors (AAV9 serotype) containing the CircSLC8A1 sequence (AAV‐Circ), and AAV‐NC were constructed and packaged by HanBio Company (China). Grouping methods of mice (each group includes six samples): Sham group, PPOL group, PPOL+AAV‐NC group and PPOL+AAV‐Circ group. A total of 10 μl of CircSLC8A1 or control vector were progressively injected into the articular cavity (about 10^6^ plaque forming units). Four weeks later, we had a second injection, and the right side of the knee joint was collected and submitted for a thorough examination 8 weeks following the initial injection.

### 2.16 Micro‐computed tomography examination

A SkyScan1276 micro‐CT (Burker Belgium) was used for the micro‐computed tomography examination of the right knee joint. The following scanning settings were chosen: Voltage (kV) = 55, Current (μA) = 72, Pixel size (μm) = 4, Filter = 0.25 mm. Dataviewer 1.013 System was used for parameter evaluation and quantification of the area of interest (ROI). Including bone mineral density (BMD), trabecular bone number (Tb.N) and bone volume per tissue volume (BV/TV).

### 2.17 Haematoxylin and eosin Staining

Tissues were put into the embedding box and washed in running water for 2 h. After dehydration, the samples were embedded in paraffin and placed on a slicer. The slicing thickness was adjusted to 5 μm. The sections were stained with haematoxylin and eosin (H&E) for 1 min and then washed away the residual staining liquid on the surface with distilled water. Images were acquired by the Olympus BX46 microscope.

### 2.18 Immunohistochemistry

The slides were washed twice in TBS solution containing 0.025% Triton X‐100 and sealed at room temperature with TBS solution containing 10% normal serum and 1% BSA for 2 h. After removing the liquid, sections were incubated with primary antibody against RUNX1 at 4°C overnight and then subjected to TBS solution containing 0.3% H_2_O_2_ for 15 min. The secondary antibody was diluted with TBS solution containing 1% BSA, and the horseradish peroxidase coupled secondary antibody was coated on the slide at room temperature for 1 h. Haematoxylin was used for counterstain.

### 2.19 Statistical analysis

GraphPad Prism 9 and SPSS 22.0 were applied to conduct statistical analysis on all data sets, which were expressed as mean ± SD. The Shapiro–Wilk test was used to determine the variability of the data distribution, and the Levene's test was used to determine the equality of variances. To compare two groups statistically, the unpaired two‐tailed Student's *t*‐test and the Welch *t*‐test were utilized. A one‐way analysis of variance was used to compare data from many groups. For all analyses, group differences greater than 0.05 (*p <* 0.05) were deemed statistically significant.

## 3 RESULTS

### 3.1 Characterization and expression analysis of CircSLC8A1

**FIGURE 1:**
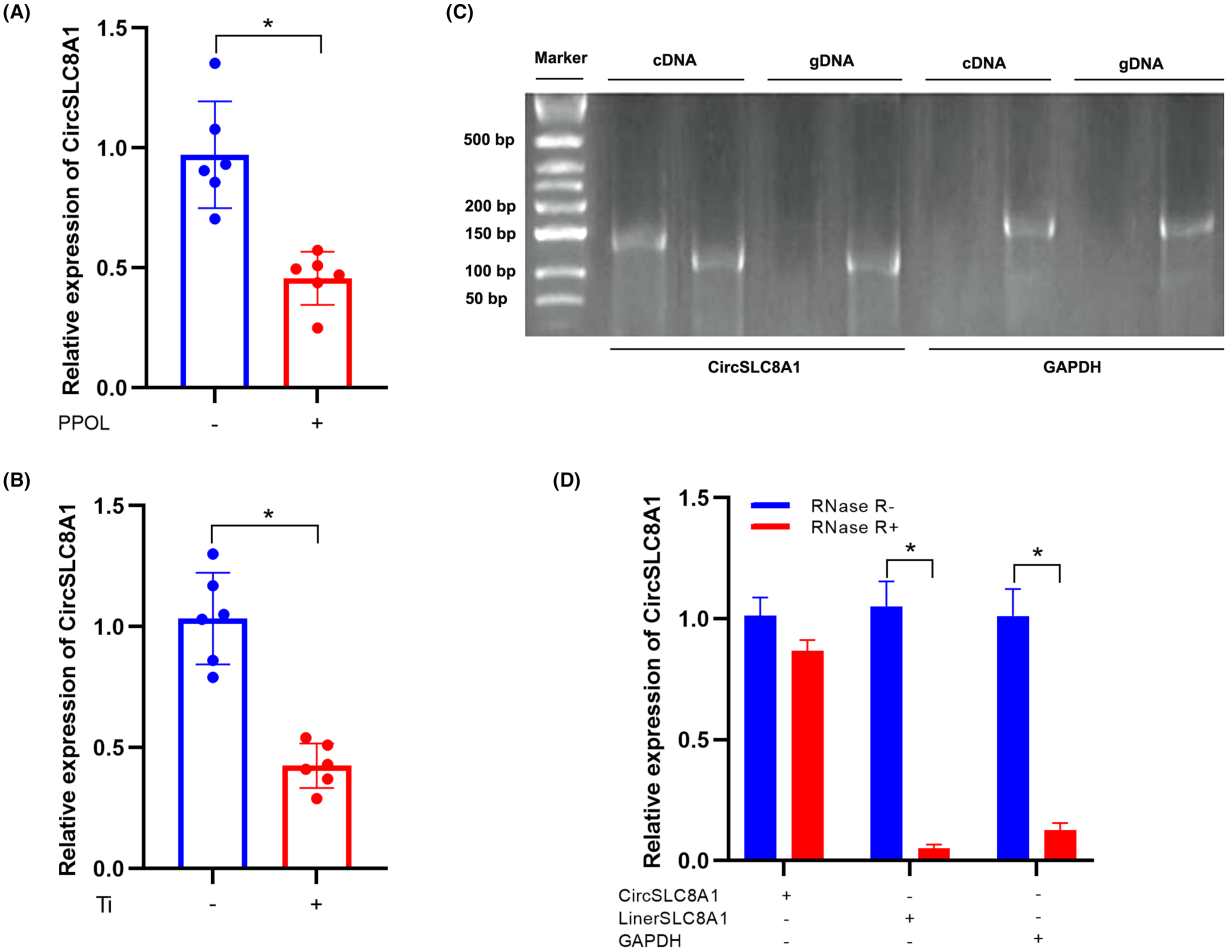
CircSLC8A1 expression and validation in patient's tissues and hBMSCs. (A) The CircSLC8A1 expression measured by qRT‐PCR was lower in PPOL patients than control group. *n* = 6; *p < 0.05. (B) The expression of CircSLC8A1 in hBMSCs treated with Ti particles (0.1 mg/mL) for 24 h. *n* = 6; *p < 0.05. (C) PCR analysis for CircSLC8A1 and SLC8A1 mRNA in cDNA and gDNA. Divergent primers amplified CircSLC8A1 from cDNA, but not from gDNA. (D) The expression of CircSLC8A1 and SLC8A1 mRNA in hBMSCs was detected by qRT‐PCR in the presence or absence of RNase R.

According to our prior report, high‐throughput sequencing technologies demonstrated that CircSLC8A1 was decreased among individuals of PPOL.^12^ In this study, another six pairs of PPOL and normal samples were analysed using qRT‐PCR to check the expression levels of CircSLC8A1 in order to confirm the observations. The results displayed a similar pattern: The expression levels of CircSLC8A1 in PPOL patients were considerably lower than in the control group (Figure 1A). Meanwhile, we employed Ti particles to act as stimulators of PPOL cell model in vitro and discovered that CircSLC8A1 expression was downregulated following treatment, which was similar to the in vivo data (Figure 1B). The samples were then submitted to PCR and agarose gel electrophoresis experiments, with convergent primers for liner SLC8A1 and special divergent primers for CircSLC8A1. CircSLC8A1 was only found in cDNA, with no products being discovered in the isolated gDNA, according to the findings (Figure 1C). Furthermore, qRT‐PCR results revealed that CircSLC8A1 was more resistant to RNase R treatment than the linear form of SLC8A1 (Figure 1D). Overall, these findings demonstrated that CircSLC8A1 was dramatically suppressed and that this was not due to chromosomal rearrangements or PCR artefacts.

### 3.2 Expression of CircSLC8A1 is increased during hBMSCs osteogenic differentiation

**FIGURE 2:**
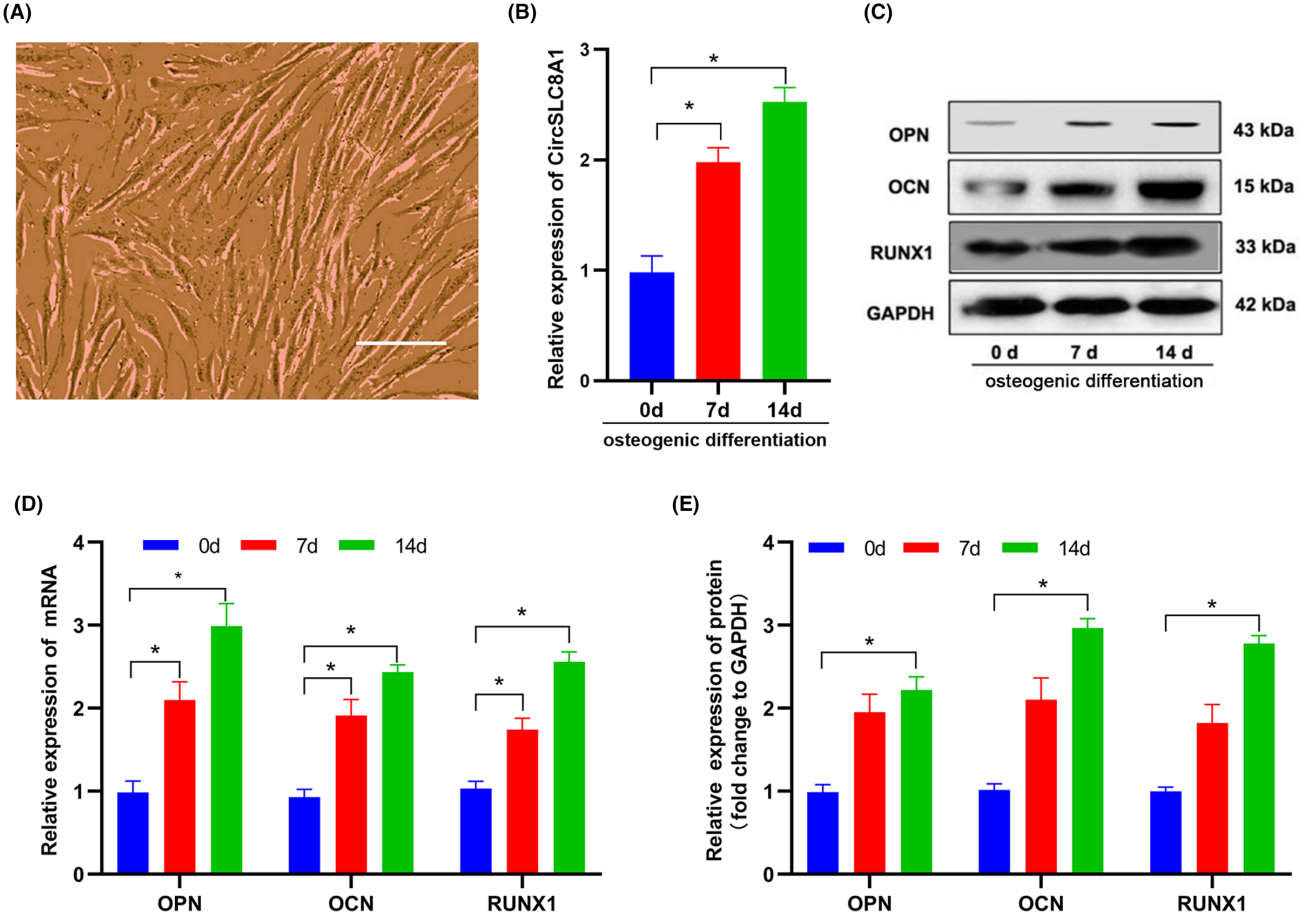
CircSLC8A1 is up‐regulated in hBMSCs during osteogenic differentiation. (A) hBMSCs were examined under a microscope. (B) CircSLC8A1 expression after treating with osteogenic differentiation medium for 0, 7, and 14 days. *n* = 3; *P < 0.05. (C, E) Western blot analysis of RUNX1, OPN and OCN expressions in hBMSCs treated with osteogenic differentiation medium for 0, 7, and 14 days. *n* = 3; *p < 0.05. (D) qRT‐PCR analysis of RUNX1, OPN, and OCN mRNA expression in hBMSCs osteogenic differentiation medium for 0, 7, and 14 days. *n* = 3; *p < 0.05

In order to better understand CircSLC8A1's role in PPOL, we conducted experiments to explore the expression and roles of CircSLC8A1 throughout osteogenic differentiation. hBMSCs were cultured in medium and collected for further passages when they achieved 80% confluency. Fibroblast‐like cells having flat or spindle‐shaped bodies could be seen in the third generation of hBMSCs (P3; Figure 2A). Furthermore, CircSLC8A1 expression in hBMSCs was detected using qRT‐PCR at certain days (0, 7, and 14 days) following osteogenic differentiation. During prolonged osteogenic differentiation in hBMSCs, CircSLC8A1 levels in the cells increased progressively (Figure 2B). Meanwhile, relative mRNA expression and protein levels of RUNX1, OPN and OCN also steadily increased during this period (Figure 2C‐E). These proofs recommend that CircSLC8A1 may act as a regulatory molecule and participate in osteogenic differentiation.

### 3.3 Effects of CircSLC8A1 on osteogenic differentiation and apoptosis

**FIGURE 3:**
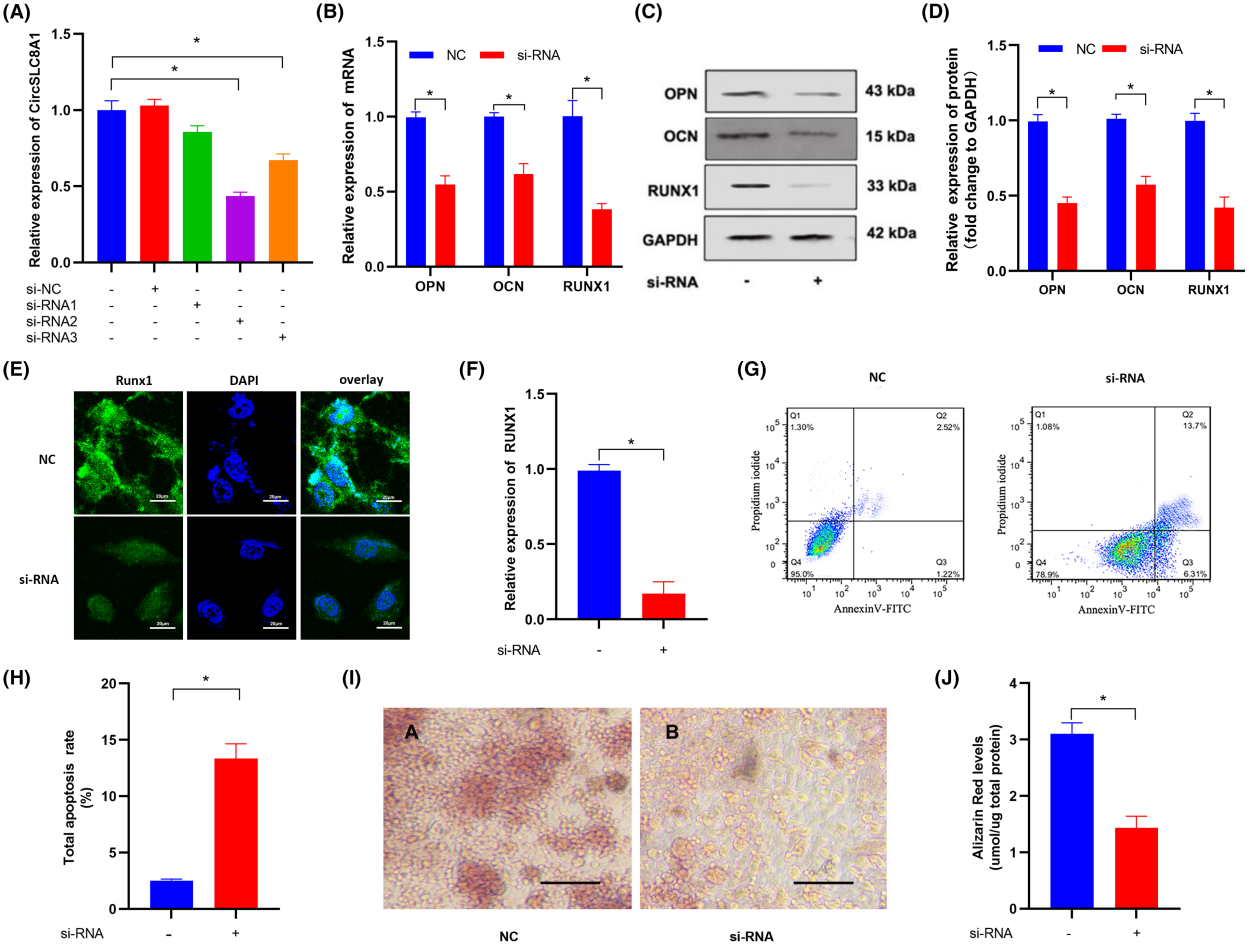
Silencing CircSLC8A1 inhibited hBMSCs osteogenic differentiation. (A) The expression level of CircSLC8A1 was measured by RT‐qPCR after hBMSCs were transfected with CircSLC8A1 siRNA or negative control siRNA for 48 h. *n* = 3; *p < 0.05. (B) qRT‐PCR analysis of RUNX1, OPN and OCN mRNA expression in hBMSCs after transfection with CircSLC8A1 siRNA or negative control. (C,D) WB analysis of osteogenic markers RUNX1, OPN and OCN expressions in hBMSCs after transfection with CircSLC8A1 siRNA or negative control. (E,F) IF of RUNX1 in hBMSCs after transfection with CircSLC8A1 siRNA or negative control. (G,H) cell apoptosis was measured by quantitative FACS analysis. *n* = 3; *p < 0.05. (I,J) hBMSCs treated with CircSLC8A1 siRNA or negative control were stained with alizarin red S to show mineralization ability. *n* = 3; *p < 0.05

**FIGURE 4:**
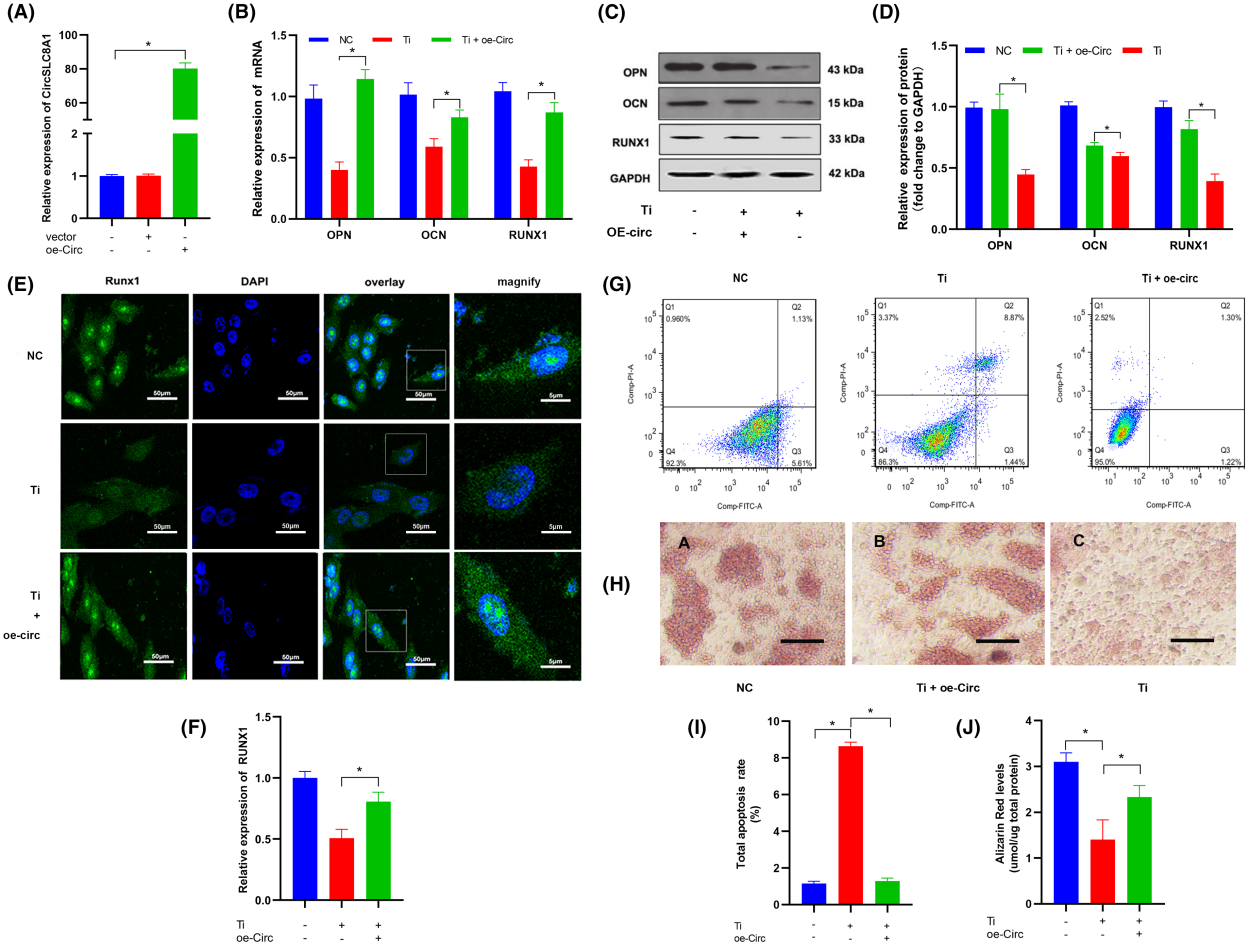
CircSLC8A1 inhibited hBMSCs osteogenic differentiation. (A) hBMSCs were transfected with CircSLC8A1 overexpression plasmid vectors or control vectors or negative control. CircSLC8A1 was detected by qRT‐PCR. *n* = 3; *p < 0.05. (B) qRT‐PCR analysis of RUNX1, OPN and OCN after hBMSCs were transfected with CircSLC8A1 overexpression plasmid vectors or negative control, followed by treatment or no treatment with Ti particles. *n* = 3; *p < 0.05. (C,D) and (E,F) WB and IF after hBMSCs were transfected with CircSLC8A1 overexpression plasmid vectors or negative control, followed by treatment or no treatment with Ti particles. (G,I) Cell apoptosis was measured by quantitative FACS analysis after CircSLC8A1 overexpression plasmid vectors or negative control, followed by treatment or no treatment with Ti particles. *n* = 3; *p < 0.05. (H,J) Alizarin red S staining detected hBMSCs mineralization ability. *n* = 3; *p < 0.05

Loss‐ and gain‐of‐function experiments in hBMSCs were used to further explore the biological functions of CircSLC8A1 in PPOL progression. Three siRNAs were generated that particularly bind the back splicing region to reduce the expression of CircSLC8A1 in hBMSCs. As a result, si‐RNA2 had better silencing efficiency and was chosen for following research (Figure 3A). The effects of CircSLC8A1 inhibition in hBMSC during osteogenic differentiation were investigated. Relatively, mRNA expression levels of RUNX1, OPN and OCN were markedly reduced as well after silencing CircSLC8A1(Figure 3B). WB and IF analysis indicated decreased protein levels of RUNX1, OPN, OCN at the same time (Figure 3C‐F). Furthermore, alizarin red S staining (ARS) revealed that knockdown of CircSLC8A1 greatly inhibited the capacity of hBMSCs to mineralize (Figure 3I,J). Quantitative fluorescence‐activated cell sorting (FACS) showed that downregulation of CircSLC8A1 increased the apoptosis rate of hBMSCs (Figure 3G,H). CircSLC8A1 was overexpressed in hBMSCs for the gain‐of‐function assay by transferring overexpression plasmid vectors. Subsequently, we investigated the effects of CircSLC8A1 in the Ti particles‐induced PPOL cell model. qRT‐PCR results confirmed that the expression level of CircSLC8A1 was elevated after OE‐circ transfected in hBMSCs (Figure 4A). Ti particles treatment increased apoptosis in hBMSCs, but overexpression of CircSLC8A1 greatly reduced the induced apoptosis, according to FACS data (Figure 4G,I). Furthermore, qRT‐PCR, WB and IF investigations revealed that following Ti particles treatment, CircSLC8A1 overexpression dramatically increased the expression of RUNX1, OPN, and OCN (Figure 4B‐F). ARS staining illustrated a reduction in the calcium nodule concentration in the cell model group as compared to the control group. To a certain extent, the opposite impact could be reversed by overexpression of CircSLC8A1 (Figure 4H,J). These findings indicate that CircSLC8A1 promotes osteogenic differentiation and reduces apoptosis.

### 3.4 CircSLC8A1 performs biological functions by sponging miR‐144‐3p

**FIGURE 5:**
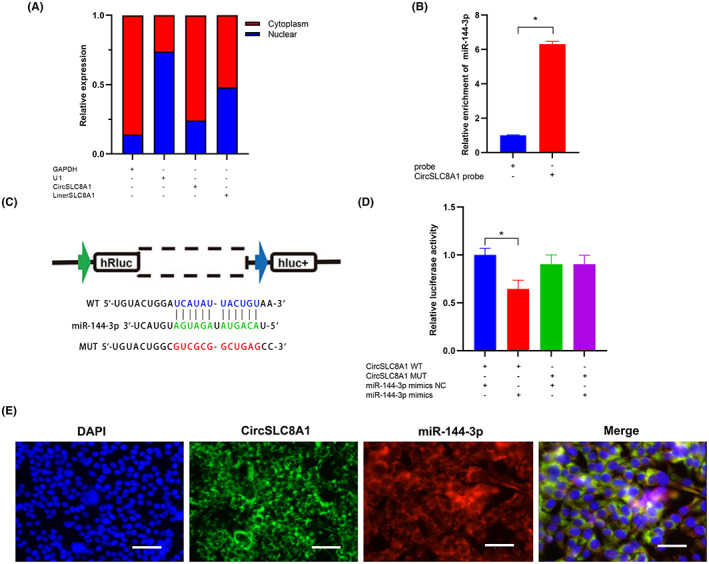
CircSLC8A1 efficiently sponges miR‐144‐3p in hBMSCs. (A) The relative expression of CircSLC8A1 and SLC8A1 mRNA in the cytoplasm and nucleus of hBMSCs was determined by qRT‐PCR and normalized to GAPDH and U1 level. *n* = 3. *p < 0.05. (B) RNA pull‐down assays were used to verify the association of CircSLC8A1 with miR‐144‐3p in hBMSCs. *n* = 3. *p < 0.05. (C) Schematic illustration demonstrating a complement to the miR‐144‐3p seed sequence with CircSLC8A1. The red letters indicate mutated nucleotides. (D) Luciferase reporter activities after miR‐144‐3p mimic or NC were cotransfected with a luciferase reporter construct containing CircSLC8A1 wt or CircSLC8A1 mut into HEK‐293T. *n* = 3; *p < 0.05. (E) FISH images showing the co‐localization of CircSLC8A1 and miR‐144‐3p in hBMSCs

**FIGURE 6:**
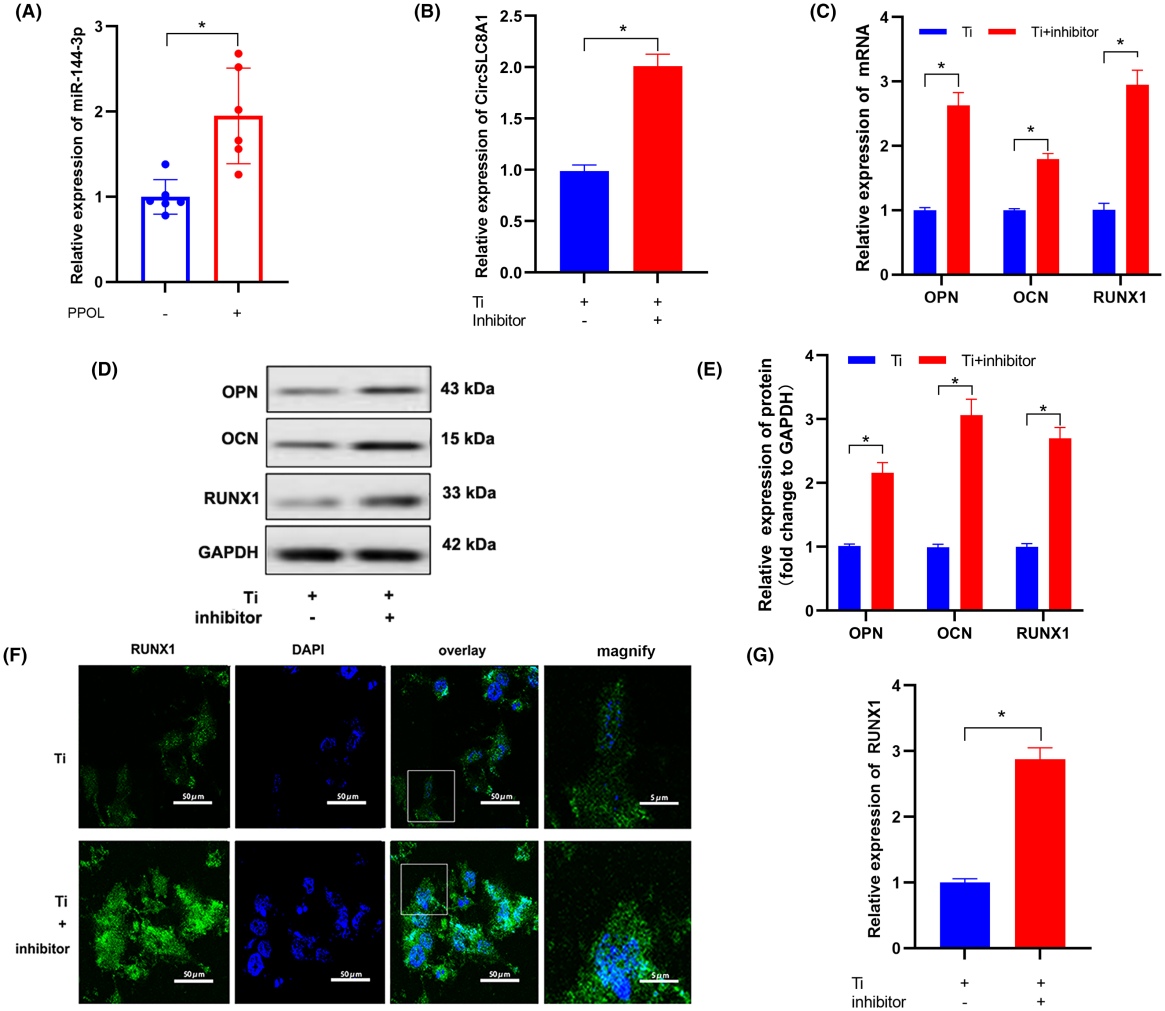
miR‐144‐3p inhibitor promotes hBMSC osteogenic differentiation. (A) The miR‐144‐3p expression was higher in PPOL patient tissues than in control group, as determined by qRT‐PCR. *n* = 6; *p < 0.05. (B) qRT‐PCR analysis of CircSLC8A1 in hBMSCs after transfection with miR‐144‐3p inhibitor. *n* = 3; *p < 0.05. (C) qRT‐PCR analysis of RUNX1, OPN and OCN mRNA expression and in hBMSCs after transfection with miR‐144‐3p inhibitor. *n* = 3; *p < 0.05. WB (D,E) and IF (F,G) after hBMSCs were transfected with miR‐144‐3p inhibitor. *n* = 3; *p < 0 0.05

The distribution of RNA in cells potentially affects its molecular mechanisms, so we investigated whether CircSLC8A1 could perform as a sponge for special miRNA if they were predominantly observed in the cytoplasm. According to the qRT‐PCR analysis with nuclear and cytoplasmic samples, CircSLC8A1 was mostly found in the cytoplasm. (Figure 5A). To further study the mechanism, circular RNA interactome database was used to predict the CircSLC8A1 interacting miRNAs. We found that CircSLC8A1 has a complementary sequence with miR‐144‐3p. To demonstrate bioinformatics prediction analysis, we conducted an RNA pull‐down assay using a biotin‐labelled CircSLC8A1 probe. When qRT‐PCR findings were compared between the CircSLC8A1 probe group and the control probe group, it was observed that miR‐144‐3p was significantly enriched in the CircSLC8A1 probe group. (Figure 5B). Finally, we constructed wild‐type vectors of CircSLC8A1 and mutant‐type vectors of CircSLC8A1 (Figure 5C). The luciferase activity of the CircSLC8A1‐WT group was substantially decreased after cotransfected with miR‐144‐3p mimics compared with NC group; but luciferase activity between CircSLC8A1‐MUT group and NC group with miR‐144‐3p mimics was not significant, suggesting a direct interaction between CircSLC8A1 and miR‐144‐3p (Figure 5D). Furthermore, CircSLC8A1 and miR‐144‐3p colocalized in the cytoplasm of hBMSCs, according to a FISH analysis (Figure 5E). Taken together, CircSLC8A1 acted as a sponge for miR‐144‐3p. We next discussed whether CircSLC8A1 participates in PPOL via interaction with miR‐144‐3p. The expression level of miR‐144‐3p was significantly greater in PPOL individuals than in the control group, as verified by qRT‐PCR (Figure 6A). Furthermore, after transferring the miR‐144‐3p inhibitor, CircSLC8A1 expression was enhanced by nearly twofold (Figure 6B). RUNX1, OPN and OCN expression were elevated at both mRNA and protein levels (Figure 6C‐G). These findings show that CircSLC8A1 performs biological tasks in hBMSCs through sponging miR‐144‐3p.

### 3.5 miR‐144‐3p directly targets RUNX1

**FIGURE 7:**
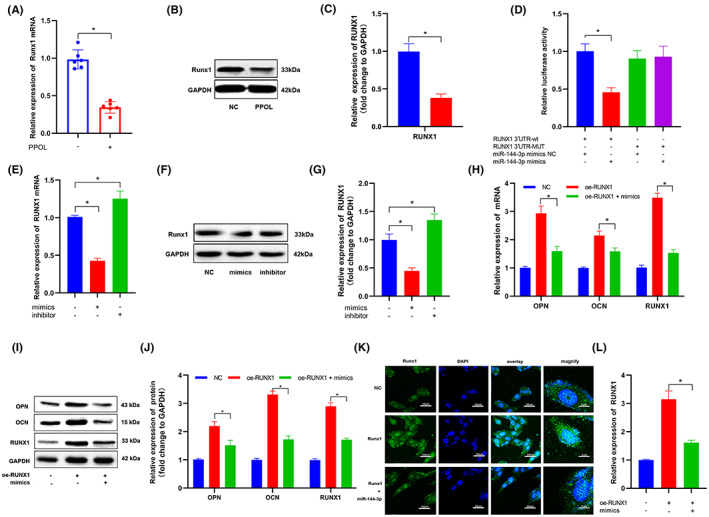
RUNX1 is directly targeted by miR‐144‐3p in hBMSCs. RUNX1 expression levels were lower in PPOL patient tissues than in control group, as determined by qRT‐PCR (A) and WB (B,C). *n* = 6; *p < 0.05. (D) HEK‐293 T cells were cotransfected with miR‐144‐3p mimics or NC and luciferase reporter constructs containing RUNX1 3′‐UTR wild‐type or RUNX1 3′‐UTR mutant. *n* = 3. *p < 0.05. hBMSCs were transfected with NC or miR‐144‐3p mimic/inhibitor, and expression level of RUNX1 were evaluated by qRT‐PCR (E) and WB (F,G). *n* = 3. *p < 0.05. (H) qRT‐PCR analysis of RUNX1, OPN and OCN mRNA expression in hBMSCs after transfection with RUNX1 overexpression plasmid vectors and miR‐144‐3p mimics. *n* = 3. *p < 0.05. WB (I,J) and IF (K,L) after hBMSCs were transfected with RUNX1 overexpression plasmid vectors and miR‐144‐3p mimics. *n* = 3. *p < 0.05

The Targetscan data set is used to analyse the downstream targets. The miR‐144‐3p complementary segment in the 3′‐UTR of RUNX1 mRNA was prospected and showed high sequence conservation. RUNX1 was chosen to be studied further. RUNX1 expression was significantly reduced in hBMSCs from PPOL (Figure 7A‐C), suggesting that RUNX1 may play a role in PPOL. miR‐144‐3p mimics were cotransfected into HEK293T cells with vectors carrying binding site sequences of RUNX1 or mutant regions. The addition of miR‐144‐3p mimics dramatically reduced luciferase activity in HEK‐293 T cells transfected with RUNX1 wild‐type, but had no statistical difference in the mutant‐type (Figure 7D). qRT‐PCR and WB analysis further demonstrated that miR‐144‐3p mimics reduced RUNX1 expression in hBMSCs, but miR‐144‐3p inhibitors boosted it (Figure 7E‐G). We cotransfected miR‐144‐3p mimics and RUNX1 overexpression vectors into BMSCs to see whether miR‐144‐3p influences the pathogenic process of PPOL via targeting RUNX1. The transfection of miR‐144‐3p mimics reversed the upregulation of RUNX1 caused by RUNX1 overexpression, according to the findings. Furthermore, whereas overexpression of RUNX1 increased OPN and OCN expression, cotransfection with miR‐144‐3p mimics partially reversed these effects (Figure 7H‐L). These findings imply that miR‐144‐3p modulates RUNX1's impact on osteogenic development by targeting it directly.

### 3.6 CircSLC8A1 improves osteogenesis and alleviates PPOL in vivo

**FIGURE 8:**
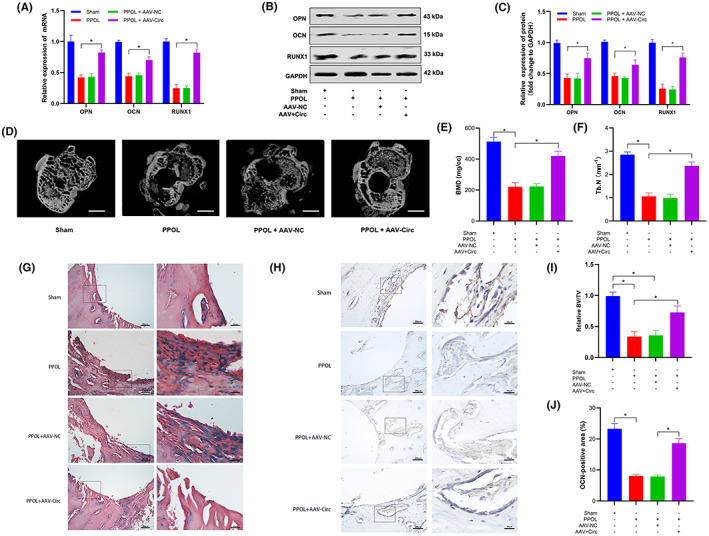
CircSLC8A1 alleviates PPOL in vivo. (A) qRT‐PCR analysis of RUNX1, OPN and OCN mRNA expression of the right knee tissues from different mouse models. *n* = 6; *p < 0.05. (B,C) WB analyses of the right knee tissues from different mouse models. (D) micro‐CT imaging for morphological structure in the knee of PPOL mice at 8 weeks after the first AAV injection, followed by treatment with intra‐articular injection of AAV‐CircSLC8A1 mouse homologous circRNA (mmu_circ_0000823) or other control groups. (E,F,I) Quantitative analysis of the corresponding parameters, including BMD, Tb.N, and BV/TV. *n* = 6; *p < 0.05. (G) H&E staining and (H,J) immunohistochemical staining for OCN of the defect area. *n* = 6; *p < 0.05

To evaluate the biological function in PPOL‐mouse mode, AAV‐mmu_circ_0000823 (mouse homologous circRNA with sequences that are 88% identical to CircSLC8A1 in humans) or an NC sequence were injected intraarticularly. There was a substantial decrease in the expression of RUNX1, OPN and OCN in the PPOL group compared with the Sham group, and there was no significant difference after AAV‐NC treatment in both protein and mRNA level. While overexpression of mmu_circ_0000823 restored the downregulated expression of RUNX1, OPN and OCN (Figure 8A‐C). Micro‐CT and quantitative analysis demonstrated that there existed an obvious bone loss in the PPOL group (Figure 8D). The parameters of BMD, Tb.N, and BV/TV were significantly decreased compared to those in the sham group. While injection of mmu_circ_0000823 could considerably alleviate osteolysis in the mouse mode (Figure 8E,F,I). HE staining results showed extensive periprosthetic interface membranes with numerous inflammatory cell infiltrations in the PPOL group. In the AAV‐Circ group, the degree of inflammatory infiltration was eased (Figure 8G). Moreover, immunohistochemical staining showed an inhibition of OCN expression in the PPOL group, which was attenuated following the injection of AAV‐mmu_circ_0000823 (Figure 8H,J). Taken together, these findings show that CircSLC8A1 acts as a promoter of osteogenic differentiation and relieves POLL in mice.

## 4 DISCUSSION

Periprosthetic osteolysis remains as the main long‐term complication following arthroplasty and seriously affects the service life of artificial joint prostheses. Both mechanical and biological elements contribute to the pathological process of PPOL. Micromotion, one of the most important mechanical factors, may result in insufficient periosteal bone formation and inadequate bone ingrowth at the bone–prosthesis interface. Stress block occurs in the proximal femur, leading to a thin cortical wall and decreased bone density, which is another factor involved in the progression.^14^ Meanwhile, biological theories emphasize the fundamental source of bone osteolysis as implant‐derived wear particles, as suggested by the established model of aseptic loosening. One of the most commonly proposed theories is that macrophages stimulated by wear particles produce chemokines and pro‐inflammatory cytokines, resulting in osteoclastic bone resorption and ultimately PPOL.^15^ However, the balance of bone turnover in the peri‐implant tissues is determined not only by osteoclast‐mediated bone resorption, but also by osteoblast‐mediated bone formation. Moreover, not only osteoclast‐mediated bone resorption but also osteoblast‐mediated bone formation affects the ratio of bone turnover in the periprosthetic tissues. In physiological environment, these two processes keep the body in a state of homeostasis. Therefore, osteoblast cell lines have become increasingly essential, and multipotent mesenchymal stem cells residing in bone marrow serve as the progenitor of these cells in the human body.^16^

Bone marrow mesenchymal stem cells (BMSCs) in periprosthetic bone and adjacent to implants promote osteogenesis and play a vital role in maintaining osseous tissue integrity. Submicron‐sized UHMWPE particles have been shown to inhibit the osteogenic differentiation of murine BMSCs in a dose‐dependent manner, as suggested in a murine model.^17^ Incubation of BMSCs with titanium (Ti) particles also caused impaired osteogenic activity and enhanced apoptosis.^18^ Another more in‐depth study compared the impact of various wear particles (Ti, PMMA, UHMWPE and Co‐Cr) on the differentiation and functions of BMSCs. Each type of particle exhibited varying degrees of suppressive effects on osteogenesis from BMSCs through the regulation of multiple gene expression associated with osteogenesis or osteoblast differentiation.^19^ Specifically, Ti and Co‐Cr particles had stronger suppression of osteogenic differentiation than PMMA and UHMWPE; UHMWPE and Co‐Cr particles contributed to an apparent increase in the pro‐inflammatory cytokine expressions, including IL‐1, IL‐6 and TNFα.

Up to now, a few of signalling pathways have been found participating in PPOL induced by wear particle‐activated BMSCs. Lin et al. presented indirect evidence that the NF‐B signalling pathway is engaged in the suppression of osteogenesis in BMSCs treated with UHMWP particles.^20^ In murine BMSCs, Ping et al. found that Ti‐particles enhanced the expression of DKK1, a Wnt/β‐catenin signalling pathway blocker, while reducing the expression of ‐catenin, suggesting that Wnt/β‐catenin signalling is involved in the suppression of osteogenic differentiation.^21^ Lee et al.^22^ pointed out that hBMSCs and other osteoprogenitors are capable of phagocytosing wear particles and initiating inflammatory responses by ERK_CEBP/β intracellular signalling pathway, which leads to subsequent accumulation of macrophages and osteoclastogenesis. Although previous research has demonstrated that diverse intracellular signalling pathways mediate the negative effects of wear debris on BMSCs, but profound studies are required to comprehend the fundamental mechanism of different nano‐sized wear articles.

In this study, we confirmed that CircSLC8A1 is suppressed in hBMSCs with titanium particles‐induced PPOL, while it was upregulated during osteogenic differentiation. To verify these results, we performed loss‐ and gain‐of function assays. Silencing CircSLC8A1 inhibited osteogenic differentiation, as evidenced by lower expression of RUNX1, OPN, OCN and impaired minerals accumulation, whereas overexpression of CircSLC8A1 would have the opposite effect. Therefore, we believed that CircSLC8A1 is a promoter of osteogenic differentiation in BMSCs. The occurrence of PPOL may correspond with abnormal expression of CircSLC8A1.

CircRNAs, as we all know, can interfere with miRNAs and regulate their activity by acting as competing endogenous RNAs (ceRNAs).^23^ miRNAs interact towards the 3'‐UTR of mRNA and regulate gene expression through RNA silencing and post‐transcriptional control. For this reason, we predicted miR‐144‐3p hit the target of C CircSLC8A1irc by bioinformatics analysis and confirmed that miR‐144‐3p directly bound to CircSLC8A1 in the following experiment. While miR‐144‐3p may be involved in PPOL processes, miRNAs have the ability to interact with hundreds of genes in mRNAs and perform a variety of functions in biological processes. For example, upregulated levels of miR‐144 have been observed in obese liver organoids, and it functions as a promoter of this progression by directly binding to Irg1, which can catalyse potent activators of NRF2.^24^ In osteosarcoma, miR‐144 suppresses aggressive phenotypes of tumour cells by targeting anoctamin 1 (ANO1).^25^ In colon cancer, miR‐144 inhibits cells proliferation, invasion and migration by downregulating SMAD4. Despite the fact that it is implicated in a broad range of disease processes, its role in PPOL has yet to be determined. We chose RUNX1 as the target of miR‐144‐3p in our research since it was one of the predicted targets and was confirmed in our mRNA sequence analysis. Following that, luciferase experiments revealed that miR‐144‐3p binds to RUNX1. We directly indicate that CircSLC8A1 regulates the expression of RUNX1 by competitively interacting with miR‐144‐3p.

Runt‐related transcription factor 1 (RUNX1), a member of the RUNX family, is one of the key regulatory proteins in vertebrates.^26^ RUNX1 is involved in embryogenesis, leukaemia, vascularization, immune response and tumour formation.^27^, ^28^, ^29^, ^30^, ^31^, ^32^, ^33^, ^34^, ^35^ But more importantly, RUNX1 has been also reported to assume an essential function in bone homeostasis. According to the research of osteoporosis in male including 822 volunteers aged 65 years or older, results pointed out that RUNX1 was a potential genetic regulatory of bone formation by measuring cortical bone and cancellous density in humans.^36^ David et al.^37^ found conditionally deleting RUNX1 from LysM‐positive osteoclast precursors could accelerate callus woven bone loss in vitro; meanwhile RUNX1 CKO mice had shown increased numbers and higher activity of osteoclasts during early fracture repair. In a mechanistic study, Tang et al.^38^ reported that knocking down the RUNX1 gene in mice resulted in dysplastic dorsal vertebra, femoral malformation, and primary osteoporosis owing to defective bone mineralization, and that RUNX1 is intimately associated to the OCN and RUNX2 regulatory sequences. The results of another experiment showed that RUNX1 deleted mice with normal bone formation had a significant osteoporosis phenotype at both the immature and adult phases.^39^ RUNX1 not only has appreciable effect on osteogenesis, but also involve in osteoclastic bone resorption. For BMSCs, RUNX1 could increase osteogenesis and decrease adipogenesis through upregulating Bmp7/Alk3/Smad1/Smad5/Smad8/Runx2 and WNT/β‐Catenin signalling pathways.^27^, ^39^ In the case of osteoclasts, RUNX1 is a disincentive of osteoclastogenesis,^40^, ^41^, ^42^ although the specific molecular mechanism remains still unclear. Above all, we conceive of RUNX1 as a key factor in maintaining skeletal homeostasis.

Our current work reveals how CircSLC8A1/miR‐144‐3p/RUNX1 modulates osteogenic differentiation in BMSCs, although some concerns remain unanswered. We proved the interaction of RUNX1 and OPN and OCN, and overexpression of RUNX1 results in upregulating OPN and OCN; however, we had not suggested the particular binding motifs. Since a variety of cells are subjected to wear particles in human body, it is difficult to define the main cause of osteolysis.^19^ The impact of RUNX1 in osteoclast or other cells will need further research. These findings will aid in a better understanding the role of CircSLC8A1 role in PPOL pathophysiology and its latent capacity for PPOL therapy.

## 5 CONCLUSIONS

In summary, our findings provided the hypothesis that CircSLC8A1 was obviously downregulated in hBMSCs with titanium particles‐induced PPOL. Furthermore, we also demonstrated that CircSLC8A1 exerted a regulatory role in blocking the activity of miR‐144‐3p, which participated in RUNX1 induced pathways in promoting osteogenic differentiation of hBMSCs. Hence, CircSLC8A1/miR‐144‐3p/RUNX1 may provide a potential target for prevention of PPOL.

## AUTHOR CONTRIBUTIONS

**Boning Yang:** Conceptualization (equal); methodology (equal); writing – original draft (lead). **Yu Qin:** Investigation (equal). **Ao Zhang:** Software (equal). **Penghao Wang:** Supervision (equal). **Hua Jiang:** Resources (equal). **Yunyi Shi:** Data curation (equal). **Guanchao You:** Data curation (equal); formal analysis (equal). **Dianlin Shen:** Validation (equal). **Shenghui Ni:** Resources (equal); writing – review and editing (equal). **Lei Guo:** Funding acquisition (lead); project administration (lead). **Ying Liu:** Methodology (equal).

## FUNDING INFORMATION

This study was financially supported by grants from the National Natural Science Foundation of China (No. 81971322).

## CONFLICT OF INTEREST

The authors declare that they have no competing interests.

## Data Availability

The data sets used and/or analysed during this study are available from the corresponding author upon reasonable request.
